# Associations Between Serum Bone Biomarkers in Early Breast Cancer and Development of Bone Metastasis: Results From the AZURE (BIG01/04) Trial

**DOI:** 10.1093/jnci/djx280

**Published:** 2018-02-07

**Authors:** Janet Brown, Emma Rathbone, Samantha Hinsley, Walter Gregory, Fatma Gossiel, Helen Marshall, Roger Burkinshaw, Helen Shulver, Hasina Thandar, Gianfilippo Bertelli, Keane Maccon, Angela Bowman, Andrew Hanby, Richard Bell, David Cameron, Robert Coleman

**Affiliations:** 1 Academic Unit of Clinical Oncology and Sheffield ECMC, University of Sheffield, Weston Park Hospital, Sheffield, UK; 2 Leeds Institute of Cancer and Pathology, University of Leeds, Leeds, UK; 3 Calderdale and Huddersfield NHS Foundation Trust, Huddersfield, UK; 4 Clinical Trials Research Unit, Leeds Institute of Clinical Trials Research, University of Leeds, Leeds, UK; 5 Academic Unit of Bone Metabolism, Metabolic Bone Centre, University of Sheffield, Northern General Hospital, Sheffield, UK; 6 Royal Surrey County Hospital, Guildford, UK; 7 Singleton Hospital, Swansea, UK; 8 Cancer Trials Ireland, University College Hospital, Galway, Ireland; 9 University of Edinburgh Cancer Research Centre, Western General Hospital, Edinburgh, UK; 10 Deakin University, Geelong, Australia

## Abstract

**Background:**

Adjuvant therapies can prevent/delay bone metastasis development in breast cancer. We investigated whether serum bone turnover markers in early disease have clinical utility in identifying patients with a high risk of developing bone metastasis.

**Methods:**

Markers of bone formation (N-terminal propeptide of type-1 collagen [P1NP]) and bone resorption (C-telopeptide of type-1 collagen [CTX], pyridinoline cross-linked carboxy-terminal telopeptide of type-1 collagen [1-CTP]) were measured in baseline (pretreatment blood samples from 872 patients from a large randomized trial of adjuvant zoledronic acid (AZURE-ISRCTN79831382) in early breast cancer. Cox proportional hazards regression and cumulative incidence functions (adjusted for factors having a statistically significant effect on outcome) were used to investigate prognostic and predictive associations between recurrence events, bone marker levels, and clinical variables. All statistical tests were two-sided.

**Results:**

When considered as continuous variables (log transformed), P1NP, CTX, and 1-CTP were each prognostic for future bone recurrence at any time (*P* = .006, *P* = .009, *P* = .008, respectively). Harrell’s c-indices were a P1NP of 0.57 (95% confidence interval [CI] = 0.51 to 0.63), CTX of 0.57 (95% CI = 0.51 to 0.62), and 1-CTP of 0.57 (95% CI = 0.52 to 0.63). In categorical analyses based on the normal range, high baseline P1NP (>70 ng/mL) and CTX (>0.299 ng/mL), but not 1-CTP (>4.2 ng/mL), were also prognostic for future bone recurrence (*P* = .03, *P* = .03, *P* = .10, respectively). None of the markers were prognostic for overall distant recurrence; that is, they were bone metastasis specific, and none of the markers were predictive of treatment benefit from zoledronic acid.

**Conclusions:**

Serum P1NP, CTX, and 1-CTP are clinically useful, easily measured markers that show good prognostic ability (though low-to-moderate discrimination) for bone-specific recurrence and are worthy of further study.

More than 40 000 women die from breast cancer annually in the United States, mainly from distant relapse, which often occurs years after initial breast cancer diagnosis (1). Bone metastases ultimately affect more than two-thirds of patients with advanced disease (2). Breast cancer cells can remain dormant for many years in the bone microenvironment, escaping the effects of adjuvant systemic therapies and retaining the potential for future activation and proliferation, resulting in metastasis in bone and/or other distant sites.

Because breast cancer cells display this affinity for bone, there is a sound rationale for targeting the bone in the adjuvant setting. Randomized trials of adjuvant bisphosphonates, confirmed by a meta-analysis of all available data (n = 18 766), have indeed shown that development of bone metastases and death from breast cancer can be reduced. However, the benefits are confined to postmenopausal patients at the time of bisphosphonate initiation (3–7), strongly suggesting that the postmenopausal bone (marrow) microenvironment has a specific interaction with tumor cell homing to bone and/or tumor dormancy.

In the AZURE trial (ISRCTN79831382) in early breast cancer, 3360 women with stage II/III breast cancer were randomized to standard adjuvant treatment alone or with the addition of zoledronic acid (zoledronate, administered over five years (3,6). With a median of 84.2 months (interquartile range [IQR] = 66–93 months) of follow-up, zoledronate improved invasive disease–free survival (IDFS) in women who were more than five years postmenopausal at diagnosis (n = 1041, adjusted hazard ratio [HR] = 0.77, 95% confidence interval [CI] = 0.63 to 0.96). Baseline (pretreatment) serum samples were collected in a subset of patients, and these provide the opportunity for prespecified analyses of relationships between bone metabolism, as determined by serum bone turnover markers and disease outcomes with or without zoledronate. The role of bone turnover markers has been extensively studied in established bone metastasis (8,9). In the current study, our aims were to determine whether, in early breast cancer, levels of bone turnover markers predicted either the risk of disease relapse (both in and outside bone) or the treatment benefits from zoledronate.

## Methods

### Patients

In the AZURE trial (3,6), following written informed consent, women with histologically confirmed breast cancer and either lymph node metastasis or T3/T4 primary tumor were randomly assigned to either standard adjuvant therapy (control) or standard adjuvant therapy plus intravenous zoledronate 4 mg (19 doses over five years).

At UK centers, ethics approval was obtained for this study, and participants gave additional consent for blood donation at study entry to be used for biomarker assessment. Serum samples were collected and stored under strict standard operating procedures temporarily at –20 °C or –80 °C at local centers before regular transfer to Sheffield for storage at –80 °C until central batch analysis.

### Laboratory Assays

Bone biomarkers were measured against reference standards in a fully accredited central laboratory (Metabolic Bone Unit, University of Sheffield) according to strict standard operating procedures. Personnel performing and reporting the analyses were blinded to clinical data.

#### Bone Biomarker Analysis

We measured two biomarkers of bone resorption, C-telopeptide of type-1 collagen (CTX), a measure of cathepsin-K-linked collagen breakdown, and pyridinoline cross-linked carboxy-terminal telopeptide of type-1 collagen (1-CTP), which is liberated by matrix metalloproteinases during degradation of mature type-1 collagen. Because 1-CTP is not produced through cathepsin-K-mediated bone resorption, its concentration is less affected by menopause (10). N-terminal propeptide of type-1 collagen (P1NP), released during collagen formation, is a robust and reliable measure of bone formation and was selected for this study (11). P1NP and CTX were measured using Cobas e411 automated immunoassays (Roche Diagnostic, Mannheim, Germany), and 1-CTP was measured by manual enzyme immunoassay (Orion Diagnostica UniQ ICTP EIA, Espoo, Finland).

The P1NP assay has a lower detection limit of 5 ng/mL and interassay coefficient of variation (CV) of 4.1%. Values were categorized as “high” if greater than 70 ng/mL based on advice from Roche Diagnostics. Results of 70 ng/mL or less were categorized as normal. The CTX assay has a measurement range of 0.010 to 6.00 ng/mL and an interassay CV of 4.0%. The upper limit of normal for premenopausal women (0.299 ng/mL) was used to categorize results as either “high” (>0.299 ng/mL) or “normal” (≤0.299 ng/mL). We also performed additional analyses with a higher threshold (high >0.556 ng/mL) to allow closer comparison with an earlier study by Lipton and colleagues (12). The 1-CTP assay was conducted manually with a lower detection limit of 0.3 ng/mL and an upper limit of normal of 4.2 ng/mL. The intraassay CV was 8.1% at 5.6 ng/mL, and the interassay CV was 7.6% at 4.8 ng/mL.

### Statistical Analysis

Statistical analysis (consistent with REMARK guidelines) was performed on the final AZURE analysis datalock with a median of 84.2 months (IQR = 66–93 months) of follow-up and 966 disease-free survival events (6). All analyses were performed on the intention-to-treat population using SAS version 9.2 or 9.4. Hypothesis testing was performed at the two-sided 5% level.

Cumulative incidence function (CIF) curves were used to investigate time to recurrence, as defined below. The Cox proportional hazards (PH) model was used to assess the relationships between the bone biomarkers and prognosis and treatment effect with zoledronate. The proportional hazards assumption was verified by assessing the statistical significance of the interaction of the relevant bone biomarker and time via an interaction term within the Cox model, as well as by a manual review of the CIF curves. Bone marker data were analyzed both as continuous variables (log transformed) and as categorical variables, using the prespecified high vs normal cut-points for both prognostic and predictive relationships.

The prespecified end points in the statistical analysis plan were: 1) time to bone recurrence, whether or not bone was the first recurrence (with deaths without prior bone recurrence censored in the Cox PH models and considered competing-risk events in CIF curves); 2) time to first recurrence in bone, including first recurrence being in bone only or concurrently with recurrence in another distant site (with deaths without prior recurrence and nonbone (only) first recurrences censored in the Cox PH models and considered competing-risk events in CIF curves); 3) time to first distant recurrence (with deaths without prior distant recurrence censored in the Cox PH models and considered competing-risk events in CIF curves).

Analyses were performed for all participants combined and according to menopausal status and were adjusted for minimization factors found to be statistically significant for disease outcomes in the main AZURE analyses (ie, lymph node involvement, estrogen receptor [ER] status, tumor stage, and type/timing of systemic therapy for each end point), as well as treatment allocation, where this was statistically significant in the main AZURE subgroup analyses. Analyses were also adjusted for treatment allocation when assessing the interaction of biomarkers with treatment (predictive analyses), where the interaction term is used to test for heterogeneity between the different biomarker levels. Exploratory analyses were carried out with a composite P1NP/CTX biomarker, in terms of both markers high vs not both markers high.

Harrell’s c-index was used to assess the discriminatory ability of the markers, with a value of 1 representing perfect discrimination and 0.5 being no better than chance. Confidence intervals for Harrell’s c-index were calculated as suggested by Newson (13).

## Results

### Patient Demographics and Baseline Data

Serum samples from 872 UK AZURE participants (441 control arm, 431 treatment arm) were analyzed, with a median follow-up of 84.2 months (IQR = 71.1–92.1 months). Baseline patient demographics (age, lymph node involvement, ER, progesterone receptor [PR], and human epidermal growth factor receptor 2 (HER2) status, menopausal status, systemic therapy, chemotherapy and statin use) in this test subpopulation were similar to the overall AZURE patient population (Table 1).

**Table 1.** Baseline demographics of patients in the biomarker subpopulation and overall AZURE population*

Parameter	Biomarker population	Overall study population
	(n = 872)	(n = 3359)
	No. (%)	No. (%)
Mean age, y	51.4	51.5
Lymph node status		
0	16 (1.8)	62 (1.8)
1–3	534 (61.2)	2075 (61.8)
≥4	320 (36.7)	1211 (36.1)
Unknown	2 (0.2)	11 (0.3)
T stage		
T1	285 (32.7)	1065 (31.7)
T2	427 (49.0)	1717 (51.1)
T3	131 (15.0)	456 (13.6)
T4	29 (3.3)	117 (3.5)
Histological grade		
1	66(7.6)	285 (8.5)
2	361 (41.4)	1439 (42.8)
3	428 (49.1)	1552 (46.2)
ER status		
Positive	676 (77.5)	2634 (78.4)
Negative	192 (22.0)	705 (21.0)
Unknown	4 (0.5)	20 (0.6)
PR status		
Positive	361 (41.4)	1423 (42.4)
Negative	205 (23.5)	806 (24.0)
Unknown	304 (34.9)	1119 (33.3)
HER2 status		
Positive	108 (12.4)	415 (12.4)
Negative	318 (36.5)	1251 (37.2)
Unknown/not measured	442 (50.6)	1672 (49.8)
Neo-adjuvant therapy intended	52 (6.0)	212 (6.3)
Systemic therapy		
Endocrine therapy alone	33 (3.8)	152 (4.5)
Chemotherapy alone	190 (21.8)	719 (21.4)
Endocrine therapy and chemotherapy	649 (74.4)	2488 (74.1)
Use of statins	43 (4.9)	197 (5.9)
Type of chemotherapy		
Anthracyclines	819 (93.9)	3132 (93.2)
Taxanes	178 (20.4)	775 (23.1)
Menopausal status		
Premenopausal	409 (46.9)	1504 (44.8)
≤5 y since menopause	123 (14.1)	490 (14.6)
>5 y since menopause	266 (30.5)	1041 (31.0)
Unknown	74 (8.5)	324 (9.6)

* ER = estrogen receptor; HER2 = human epidermal growth factor receptor; PR = progesterone receptor.

Comparison of IDFS outcomes for the biomarker population with the whole AZURE population showed that both the proportions of patients with an event and the hazard ratios were similar, though the confidence intervals were wider in the biomarker population due to the smaller number of patients. This similarity also applied when broken down into post- and nonpostmenopausal subgroups (Supplementary Figure 1, available online).

Baseline data for the three biomarkers (also broken down into menopausal status) revealed that the proportion of patients in each category who fall above the normal ranges for P1NP, CTX, and 1-CTP for the whole population were 27.3%, 30.0%, and 50.5%, respectively (Table 2), confirming that the data were appropriate to test the relationship between accelerated baseline bone turnover and subsequent distant recurrence events.

**Table 2.** Distribution of patients with high/normal bone marker values according to menopausal status*

Biomarker	Whole population	Premenopausal	0–5 y postmenopausal	>5 y postmenopausal
P1NP				
Assay, median (IQR), ng/mL	55.1 (41.2–72.7)	49.1 (37.3–64.3)	58.4 (42.8–76.1)	64.8 (48.1–84.4)
No. of patients	867	409	121	263
% > 70 ng/mL	27.3	18.3	30.1	38.7
CTX				
Assay, median (IQR), ng/mL	0.23 (0.15–0.32)	0.18 (0.13–0.26)	0.25 (0.18–0.37)	0.29 (0.21–0.41)
No. of patients	863	408	120	262
% > 0.299 ng/mL	30.0	17.4	37.4	45.9
% > 0.556 ng/mL	4.2	1.0	7.3	7.9
1CTP				
Assay, median (IQR), ng/mL	4.25 (3.26–5.15)	3.99 (3.12–4.95)	4.26 (3.20–5.02)	4.60 (3.77–5.41)
No. of patients	861	408	118	265
% > 4.2 ng/mL	50.5	44.5	49.6	61.7

* This table does not include data for the patients whose menopausal status was unknown, included in the whole study population. 1-CTP = pyridinoline cross-linked carboxy-terminal telopeptide of type-1 collagen; CTX = C-telopeptide of type-1 collagen; IQR = interquartile range; P1NP = N-terminal propeptide of type-1 collagen.

### Bone Biomarker Prognostic Analyses

**Figure 1.**
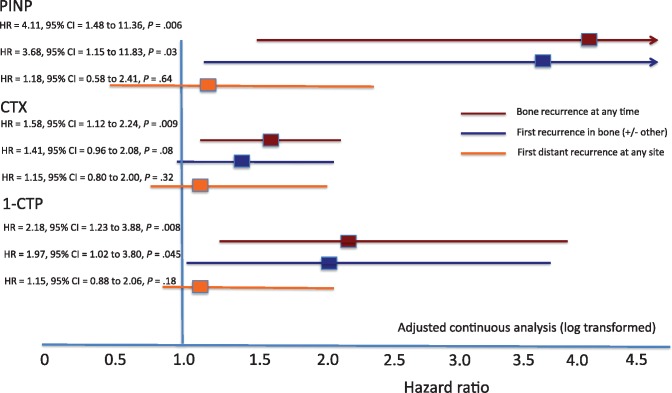
Hazard ratios and 95% confidence intervals for adjusted continuous analyses of log-transformed data for baseline N-terminal propeptide of type-1 collagen, C-telopeptide of type-1 collagen, and pyridinoline cross-linked carboxy-terminal telopeptide of type-1 collagen and disease outcomes. *P* values were calculated using the likelihood ratio χ^2^ test statistic, and tests were two-sided. 1-CTP = pyridinoline cross-linked carboxy-terminal telopeptide of type-1 collagen; CI = confidence interval; CTX = C-telopeptide of type-1 collagen; HR = hazard ratio; P1NP = N-terminal propeptide of type-1 collagen.

Figure 1 and Table 3 display key data for prognostic analyses in the three prespecified recurrence categories. The proportional hazards assumption was also investigated for each Cox proportional hazards model applied in our study. The majority of markers and end points were not close to violating this assumption (ie, suggesting no difference in the effects of the markers as time elapses). For the 1-CTP marker, the assumption was only borderline valid, suggesting that the impact of 1-CTP may differ as time elapses, although the Cox proportional hazards model was still appropriate.

**Table 3.** Adjusted analyses for high vs normal values of bone markers and analyses for interquartile range change for continuous analyses

Analysis	Bone marker high No. of events/patients (%)	Bone marker normal No. of events/patients (%)	Adjusted categorical analysis		Adjusted continuous analysis	
			HR (95% CI)	P*	HR for IQR change (log transformed) (95% CI)†	P*
PINP (70 ng/mL cut-point, n = 867)						
Bone recurrence at any time	37/238 (15.5)	67/629 (10.7)	1.61 (1.07 to 2.42)	.03	1.42 (1.10 to 1.82)	.006
First recurrence in bone	29/238 (12.2)	53/629 (8.4)	1.58 (1.00 to 2.50)	.06	1.38 (1.04 to 1.84)	.03
First distant recurrence (at any site)	54/238 (22.7)	134/629 (21.3)	0.99 (0.72 to 1.37)	.96	1.04 (0.87 to 1.24)	.64
IDFS	69/238 (29.0)	179/629 (28.5)	0.97 (0.73 to 1.29)	.83	1.06 (0.91 to1.25)	.43
CTX (0.299 ng/mL cut-point, n = 863)						
Bone recurrence at any time	40/262 (15.3)	64/601 (10.6)	1.55 (1.05 to 2.31)	.03	1.41 (1.09 to 1.82)	.009
First recurrence in bone	28/262 (10.7)	54/601 (9.0)	1.27 (0.80 to 2.00)	.32	1.29 (0.97 to 1.72)	.08
First distant recurrence (at any site)	67/262 (25.6)	121/601 (20.1)	1.26 (0.93 to 1.71)	.13	1.11 (0.92 to 1.33)	.27
IDFS	87/262 (33.2)	161/601 (26.8)	1.24 (0.96 to 1.62)	.11	1.15 (0.99 to 1.35)	.08
CTX (0.556 ng/mL cut-point, n = 863)						
Bone recurrence at any time	7/37 (18.9)	97/826 (11.7)	1.95 (0.90 to 4.21)	.12	1.41 (1.09 to 1.82)	.009
First recurrence in bone	6/37 (16.2)	76/826 (9.2)	2.17 (0.94 to 5.01)	.10	1.29 (0.97 to 1.72)	.08
First distant recurrence (at any site)	9/37 (24.3)	179/826 (21.7)	0.94 (0.47 to 1.86)	.85	1.11 (0.92 to 1.33)	.27
IDFS	14/37 (37.8)	234/826 (28.3)	1.15 (0.66 to 2.00)	.62	1.15 (0.99 to 1.35)	.08
1-CTP (4.2 ng/mL cut-point, n = 861)						
Bone recurrence at any time	59/440 (13.4)	45/421 (10.7)	1.39 (0.94 to 2.05)	.10	1.43 (1.10 to 1.86)	.008
First recurrence in bone	45/440 (10.2)	37/421 (8.8)	1.30 (0.84 to 2.02)	.24	1.36 (1.01 to 1.84)	.045
First distant recurrence (at any site)	100/440 (22.7)	87/421 (20.7)	1.19 (0.89 to 1.59)	.25	1.15 (0.94 to 1.39)	.18
IDFS	131/440 (29.8)	116/421 (27.6)	1.18 (0.91 to 1.53)	.21	1.20 (1.00 to 1.42)	.04

* *P* values were calculated using the likelihood ratio χ^2^ test statistic, and tests were performed at the two-sided 5% significance level. 1-CTP = pyridinoline cross-linked carboxy-terminal telopeptide of type-1 collagen; CI = confidence interval; CTX = C-telopeptide of type-1 collagen; HR = hazard ratio; IDFS = invasive disease–free survival; IQR = interquartile range; P1NP = N-terminal propeptide of type-1 collagen.

† These results show the hazard ratio for an interquartile range increase in the log_10_ (P1NP) or ln (CTX, 1-CTP) transformed variables. The *P* value of these analyses is unchanged from the adjusted continuous analyses shown in Figure 1.

#### Bone Recurrence at Any Time

**Figure 2.**
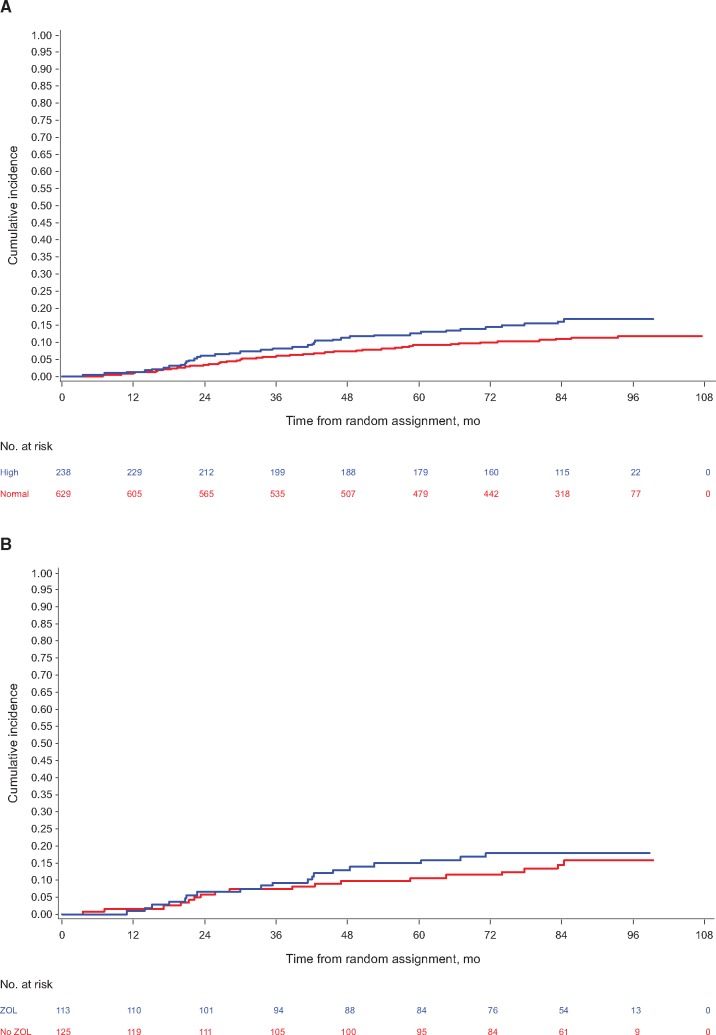
**A)** Cumulative incidence function for time to bone metastasis at any time for categorical analysis of N-terminal propeptide of type-1 collagen (P1NP) level ≥ or < 70 ng/mL (hazard ratio [HR] for adjusted analyses = 1.61, 95% confidence interval [CI] = 1.07 to 2.42, *P* = .03). **B)** Cumulative incidence function for time to bone metastasis at any time by treatment arm for participants with high P1NP (≥70 ng/mL; HR for adjusted analyses = 0.989, 95% CI = 0.517 to 1.895, *P*_interaction_ = .69 for the interaction between P1NP and treatment; ie, to assess for differing effects of treatment within the two groups of high or normal P1NP). *P* values were calculated using the likelihood ratio χ^2^ test statistic, and tests were two-sided. 1-CTP = pyridinoline cross-linked carboxy-terminal telopeptide of type-1 collagen; CTX = C-telopeptide of type-1 collagen; P1NP = N-terminal propeptide of type-1 collagen; ZOL = zoledronate 4 mg (19 doses over 5 years).

In adjusted continuous log-transformed analyses (Figure 1), increases in all three markers were strongly associated with statistically significantly increased risk of development of bone metastasis (P1NP: *P* = .006; CTX: *P* = .009; 1-CTP: *P* = .008). In categorical analyses, P1NP greater than 70 ng/mL (*P* = .03) and CTX greater than 0.299 ng/mL (*P* = .03), but not CTX greater than 0.566 ng/mL (*P* = .12) or 1-CTP greater than 4.2 ng/mL (*P* = .10), were statistically significantly prognostic for recurrence in bone at any time (Table 3). Cumulative incidence plots for categorical analysis of time to bone metastasis at any time are shown, exemplified for P1NP, in Figure 2 for both control and treatment arms.

Taking P1NP, on the basis of the above data, as the likely most sensitive prognostic factor, we tested the role of menopausal status in P1NP analyses (data not shown). However, we detected no statistically significant prognostic effect of P1NP on bone recurrence in either postmenopausal or nonpostmenopausal patients, when analyzed with P1NP as either a categorical or continuous variable.

Harrell’s c-index values (when coded as [log] continuous variables) were similar for all three markers: P1NP c-index was 0.57 (95% CI = 0.51 to 0.63); CTX c-index was 0.57 (95% CI = 0.51 to 0.62); and 1-CTP c-index was 0.57 (95% CI = 0.52 to 0.63).

#### First Recurrence in Bone (+/− Concurrent Recurrence Elsewhere)

In the adjusted continuous analyses, both P1NP (*P* = .03) and 1-CTP (*P* = .045), appeared statistically significantly prognostic for first recurrence in bone (Figure 1, Table 3). However, in adjusted categorical analyses, although the hazard ratios for each marker were similar to bone recurrence at any time, the 95% confidence intervals were wide, and no statistically significant relationships between higher marker values and first disease recurrence in bone were seen. The number of bone-only first recurrence events was too small to justify separate analysis of this potential end point of interest.

#### First Distant Recurrence (Whatever the Site)

There were no associations in either continuous or categorical analyses between baseline P1NP, CTX, or 1-CTP and development of distant recurrence at any site (Figure 1, Table 3), clearly demonstrating that, in contrast to recurrence specifically in bone, the markers were not prognostic for distant metastasis taken as a whole. Categorical data for IDFS were statistically nonsignificant.

#### Composite P1NP and CTX Biomarker Analysis

Adjusted analyses were performed to assess risks of recurrence for patients where both P1NP and CTX (using the 0.299 ng/mL cut-point) were high compared with all other patients; details are displayed in Table 4. No statistically significant relationships were identified between the composite marker and subsequent recurrence, although there was an increased risk for bone recurrence at any time in the patients with elevation of both biomarkers (HR = 1.60, 95% CI = 0.99 to 2.48, *P* = .06). Consideration was given to a joint Cox model containing all three markers, but there were insufficient events to make this meaningful. Further, 1-CTP levels represent a different aspect of the bone turnover process than P1NP and CTX and are largely unaffected by inhibitors of osteoclast function such as bisphosphonates (10).

**Table 4.** Adjusted prognostic categorical analyses according to a composite P1NP-CTX marker for both P1NP and CTX high vs not both high

Analysis	P1NP, CTX		Adjusted analysis		
	Both high events/censored (%)	Not both high events/censored (%)	HR (95% CI)		P*
Bone recurrence at any time	24/118 (16.9)	80/641 (11.1)	1.60 (0.99 to 2.48)		.06
First recurrence being in bone	18/124 (12.7)	64/657 (8.9)	1.50 (0.89 to 2.54)		.14
First distant recurrence (at any site)	34/108 (23.9)	154/567 (21.4)	1.00 (0.68 to 1.45)		.99
First recurrence being in bone only	11/131 (7.7)	47/674 (6.5)	1.26 (0.65 to 2.44)		.50

* *P* values were calculated using the likelihood ratio χ^2^ test statistic, and tests were performed at the two-sided 5% significance level. 1-CTP = pyridinoline cross-linked carboxy-terminal telopeptide of type-1 collagen; CI = confidence interval; CTX = C-telopeptide of type-1 collagen; HR = hazard ratio; IDFS = invasive disease–free survival; P1NP = N-terminal propeptide of type-1 collagen.

#### Sensitivity Analyses Assessing Optimum Cut-Points

**Figure 3.**
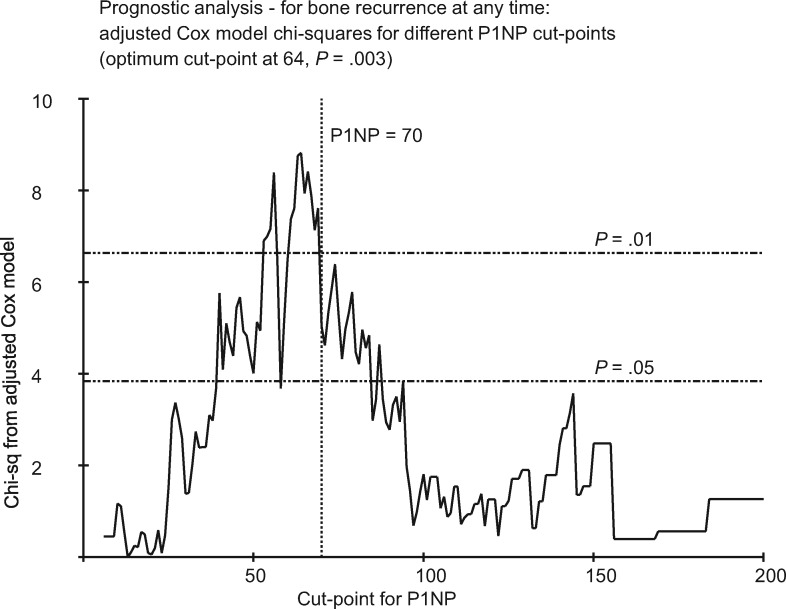
χ² values from adjusted Cox proportional hazards model, analyzing bone metastasis at any time by N-terminal propeptide of type-1 collagen (P1NP), with differing high vs normal P1NP cut-points. Optimum cut-point observed at 64 ng/mL with a corresponding *P* value of .003. *P* values were calculated using the likelihood ratio χ^2^ test statistic, and tests were two-sided. P1NP = N-terminal propeptide of type-1 collagen.

We explored the effects of different cut-points for categorical prognostic analysis of P1NP and bone metastasis at any time. This analysis (Figure 3) showed that the optimal cut-point for P1NP was approximately 64 nmol/mL, which we judged was sufficiently close to the prespecified value of 70 nmol/mL, bearing in mind that the number of events was not sufficient to generate a smooth relationship. For 1-CTP and CTX, similar exploration yielded no clearly optimal cut-point or improvement to those preselected (Supplementary Figures 2 and 3, available online).

### Analyses for Treatment Effect—Test for Predictive Biomarkers

Although P1NP is higher in postmenopausal women and the benefits of zoledronate are largely restricted to this subset of patients (6,7), baseline P1NP did not predict benefit from zoledronate when assessed against bone metastasis at any time outcome. For example, in categorical analyses considering the effect of P1NP on bone recurrence at any time, there were no statistically significant differences in outcome between the zoledronate and control arms for either high P1NP (HR = 0.99, 95% CI = 0.52 to 1.90) or normal P1NP (HR = 0.84, 95% CI = 0.52 to 1.37) with a nonsignificant *P_interaction_* value for the interaction of P1NP and treatment (*P* = .69) (Figure 1B).

We also found no statistically significant interaction with treatment allocation for either of the other defined, less frequent outcome categories with any of the bone markers, or with the P1NP/CTX composite bone marker. However, considering the complex inter-relationships between treatment effect and menopausal status in the main AZURE study, the numbers of events are likely insufficient for definitive analysis.

Corresponding continuous (log-transformed) analyses for bone metastases at any time found no statistically significant interaction with treatment allocation for any of the markers analyzed (P1NP: *P* = .74; CTX: *P* = .47; 1-CTP: *P* = .31), confirming that these baseline markers are not predictive of the treatment benefits of zoledronate.

## Discussion

Our study showed that patients with high serum levels of P1NP, CTX, or 1-CTP shortly after diagnosis of early breast cancer were associated with a higher risk of developing bone metastasis during the course of their disease. P1NP appeared to be the most sensitive of the markers studied, but was not predictive of benefit from zoledronate. Using CTX and P1NP as a composite biomarker did not add to the sensitivity of the individual markers. This may be partially because the markers are not independent, reporting on essentially linked metabolic processes, but may also be due to the relatively small number of events in the combined group.

It has been long-established that the rate of bone loss accelerates relatively rapidly in perimenopause, across the menopausal transition, with consequent increase in bone turnover markers, associated with an accelerated decrease in measured bone mineral density (BMD) (14,15). The inverse relationship between loss of BMD and increase in bone turnover markers (including P1NP and CTX) from premenopause through perimenopause to postmenopause is well established (16). As anticipated, our data reflect this pattern, with baseline bone marker values all increasing progressively from premenopause, through the early years following cessation of menses, to more than five years postmenopause.

Although all three markers and especially P1NP are good predictors of bone-specific recurrence, the calculated values of Harrell’s c-index (each around 0.57) suggest that they have only low-to-moderate discrimination, although this is statistically different from a chance finding as shown by the lower limit in the 95% confidence intervals being greater than 0.5. From a clinical perspective, however, it should be borne in mind that even for the two key prognostic indicators in everyday use in breast cancer (lymph nodes and stage), c-indices are 0.62 and 0.63, respectively (W. Gregory, personal communication), only marginally greater than those values reported for the three bone markers in this study.

Our findings are consistent with a bone microenvironment with increased bone turnover, providing a fertile “soil” for the development of skeletal metastasis. By contrast with this clear association between baseline bone turnover markers and recurrence in bone, there was no association detectable between bone turnover markers and distant recurrence taken as a whole, indicating that bone turnover markers specifically provide prognostic information for future recurrence in bone and not for metastasis more generally (17–19). We acknowledge that, in some cases, elevation of baseline bone markers may be linked with active, but as yet undetected, bone metastases. However, the relatively long follow-up (median = 84 months) and few bone events in the first two years (<5%), when the cumulative incidence curves diverge, makes it unlikely that the raised markers are simply an early diagnostic indication of bone metastases.

There is important literature evidence supporting our study. In particular, Lipton et al. (12) investigated β-CTX in 621 postmenopausal early breast cancer patients in a five-year phase III trial of tamoxifen +/− octreotide. Over 7.9 years (median) of follow-up, 19 (3.1%) patients developed bone-only recurrence as first event, 47 (7.5%) developed bone and concurrent other relapse as first event, and 57 (9.2%) developed first recurrence in sites excluding bone. Using a categorical analysis (cut-point = 0.71 ng/mL), higher pretreatment β-CTX was associated with shorter bone-only recurrence-free survival (HR = 2.8, 95% CI = 1.05 to 7.48, *P* = .03). However, there was no statistically significant association with first event in the bone plus concurrent relapse elsewhere or with first recurrence at other distant sites. It should be noted that there were differences in the patient populations and administration of bone-targeted therapy between the Lipton et al. study and our data (the former included only postmenopausal patients whose tumors were mostly ER positive with consequent lower-risk disease).

A limitation of our study is that only baseline biomarker measurements were available, although because the proportional hazards assumption was not violated, this suggests no difference in the effect of the markers as time elapses. While our data suggest that the rate of bone turnover at this early stage of disease when tumor cells may be homing to potential metastatic sites is a statistically significant contributing factor to development of bone metastasis, changes in subsequent bone turnover may also play a role. There is evidence that this might be the case in a study that assessed paired serum samples at baseline and one year within a large, placebo-controlled, randomized study of oral clodronate in early breast cancer (20). Although baseline P1NP was not prognostic for developing bone metastasis after five years of follow up, the incidence of bone metastasis was statistically significantly higher in women whose P1NP value increased by more than 20% in the first year (*P* < .02).

A further possible limitation of our analysis is that the biomarker population comprised slightly more than 25% of the total AZURE population. Although we have shown that both baseline demographics and outcomes of the biomarker population and the total trial population are similar, this cannot completely exclude the possibility of bias in the population analyzed.

Because the treatment benefits of adjuvant zoledronate in postmenopausal women might be related to inhibition of the increased bone turnover associated with menopause, our finding that baseline P1NP levels were not predictive for benefit from zoledronate was initially surprising. However, a number of factors (in addition to low event numbers in some analyses) may contribute to this result. Administration of multiple doses of a potent bisphosphonate can confidently be assumed to suppress bone turnover throughout the five-year treatment period. This could render baseline marker values less relevant in analyses of association. Also, it should be noted that, although zoledronate only produced a benefit in overall invasive relapse in patients who were five or more years postmenopause, it was associated with a reduction in first and subsequent metastasis to bone across all menopausal groups (6). Additionally, bone turnover markers reflect activity across the skeleton as a whole, whereas the amount of bone associated with disseminated tumor cells likely comprises only a very small fraction of the total skeletal metabolic activity. Finally, there is the intriguing possibility that the efficacy of zoledronate in the adjuvant setting may be due to a direct toxic effect on tumor cells in the bone microenvironment and independent of its action on bone turnover.

Other recent studies have also addressed the need for prognostic/predictive biomarkers relating to adjuvant bone-targeted treatment in early breast cancer. Using primary tumor tissue from patients in the AZURE study, we showed that a novel composite biomarker comprising the proteins CAPG and GIPC1 was prognostic for developing bone metastasis (HR = 4.5, 95% CI = 2.1 to 9.8, *P* < .001) and predicted response to zoledronate (*P* = .008) (21). In another study, amplification of the 16q23 chromosomal region, including amplification of the MAF gene (22), was predictive of breast cancer metastasis to bone (23). However, there remains a need for a simple blood-based test in early breast cancer that can identify patients with a high risk for development of bone metastasis. Bone turnover markers are easily measured and are worthy of additional investigation in helping to meet this need.

## Funding

This work was supported by Cancer Research UK (through the awards of a Research Studentship to ER, a Clinician Scientist Fellowship to JEB, and support to the Sheffield Experimental Cancer Medicine Centre) and a grant from Novartis Pharmaceuticals.

## Notes

The funders had no role in the design of the study; the collection, analysis, or interpretation of the data; the writing of the manuscript; or the decision to submit the manuscript for publication. Novartis provided academic grant support and supplies of zoledronic acid (Zometa) for the AZURE trial. The authors disclose the following: JB received fees from Novartis and Amgen for Advisory Boards and Speakers Bureaux; WG received fees from Celgene for statistical consultancy and honoraria from Janssen; DC received nonfinancial support from Novartis; RC received institutional research grants from Amgen and Bayer, fees from Novartis for expert testimony, and lecture fees from Amgen. All other authors declared no conflicts.

We wish to thank the AZURE trial patients who provided blood samples to support this research.

## Supplementary Material

Supplementary Data
