# Supplementary material for: Associations Between Serum Bone Biomarkers in Early Breast Cancer and Development of Bone Metastasis: Results From the AZURE (BIG01/04) Trial
Source: J Natl Cancer Inst. 2018 Feb 7;110(8):871–9. doi: 10.1093/jnci/djx280 (PMC6093369; doi:10.1093/jnci/djx280)

Supplementary Figure 1. Forest plot of Invasive Disease Free Survival (IDFS) treatment hazard ratios and 95% confidence intervals (CIs) for all patients in the AZURE study (black) and patients in the biomarker population (blue). P-values were calculated using the likelihood ratio χ^2 test statistic and tests were performed at the two-sided 5% significance level

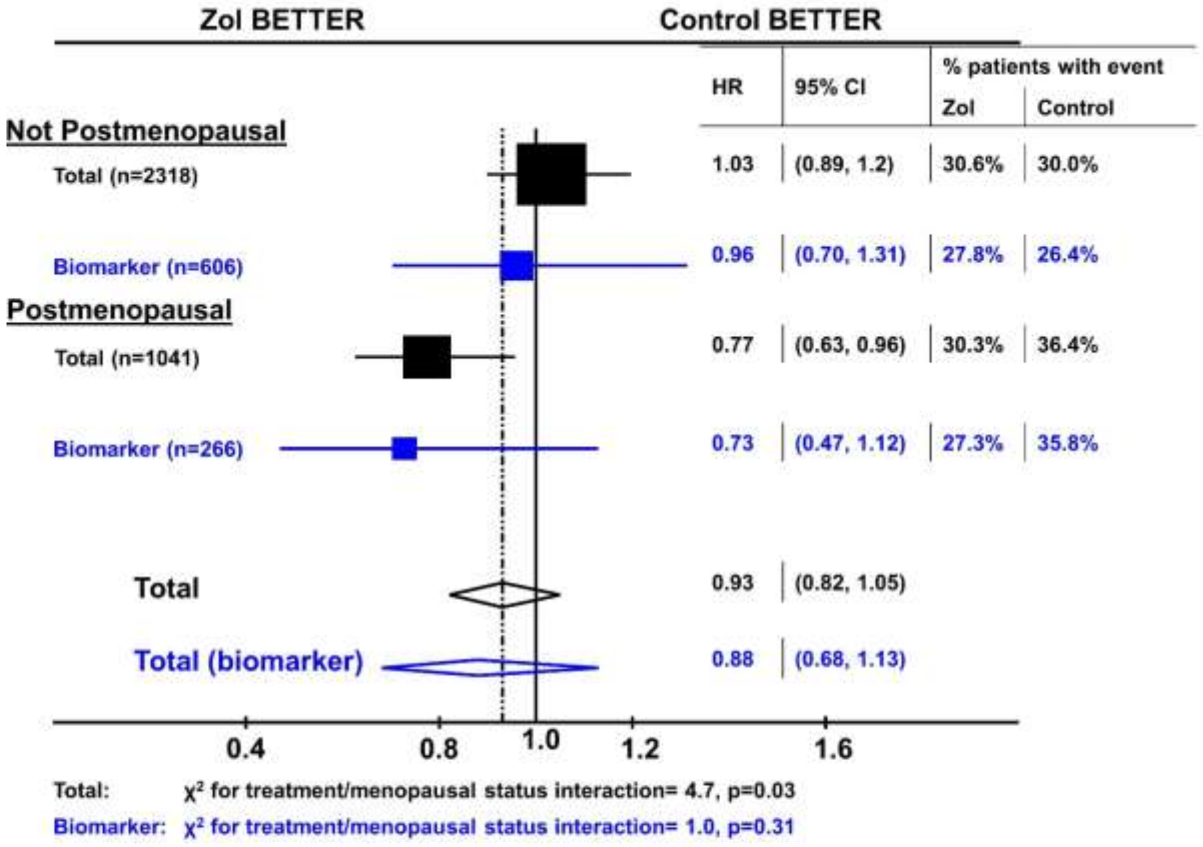

Supplementary Figure 2. χ^2 values from adjusted Cox proportional hazards model, analysing bone metastasis at any time by CTX, with differing high vs. normal CTX cut-points. Optimum cut-point observed at 0.2 ng/ml with a corresponding p-value of 0.01. P-values were calculated using the likelihood χ^2 test statistic and tests were performed at the two-sided 5% significance level.

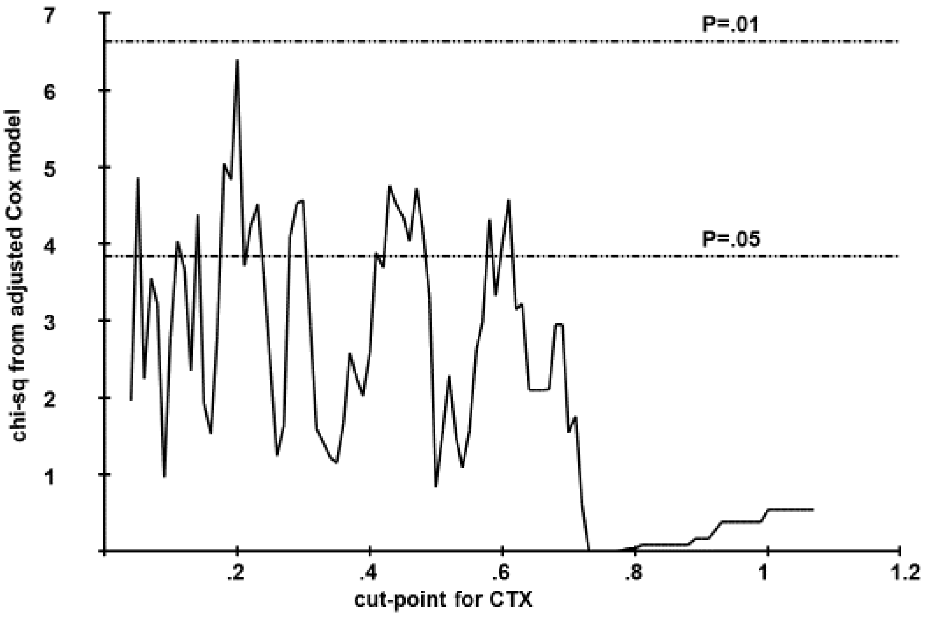

Supplementary Figure 3. χ^2 values from adjusted Cox proportional hazards model, analysing bone metastasis at any time by 1-CTP, with differing high vs. normal 1-CTP cut-points. Optimum cut-point observed at 3.7 ng/ml with a corresponding p-value of 0.01. P-values were calculated using the likelihood χ^2 test statistic and tests were performed at the two-sided 5% significance level.

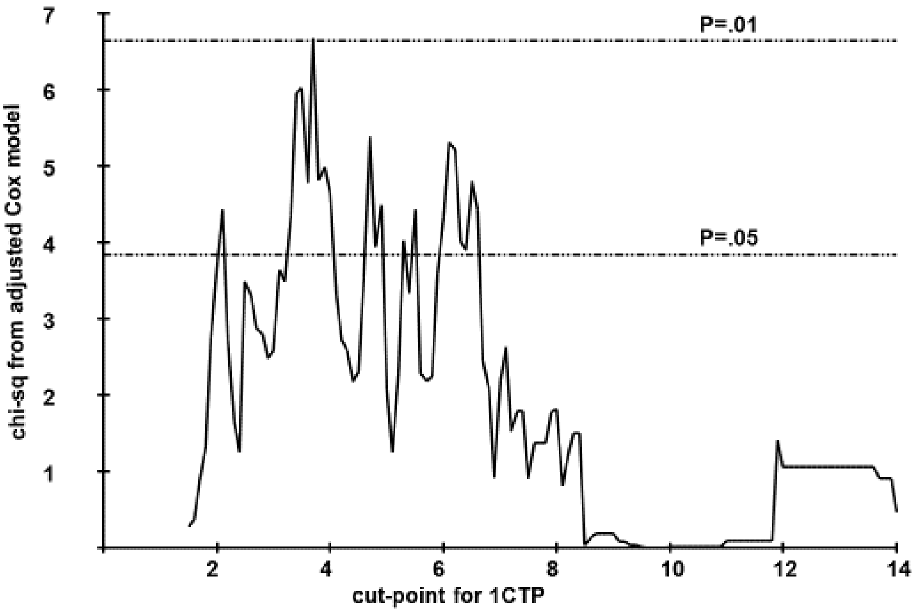

Supplement: Supplementary Data [file djx280_supp.pdf]
